# Solar Asphyxia, or Coup De Soleil

**Published:** 1845-09

**Authors:** 


*Solar Asphyxia, or Coup de Soleil.* (See New York Med. Gaz., 1841.)

July 24th, 1845. At 6 A. M., air, 80°; at 8, 85°. River, 83¾°; at 10, air 88°; at 11, 91°; at 12, 93°; at 1 P. M., near the sun, 94°; at 2, 96°; at 3, 96°; at 4, 96°. Called at 6 P. M., to a case of Solar Asphyxia, (now almost epidemic,) corner of Camp and Julia streets: George Jennings, born in Boston; aged 28;—fell 30 to 40 minutes before I saw him, and lived 20 minutes after; the experiment continued 3¼ hours, and was witnessed by many persons; the record, after the first two hours, was kept by Mr. Banks, apothecary, and by Messrs. Cochran and Vandergrif. Axilla, 20 minutes before death, continued at 111°; in 8 m. after death, 112°; in about 15 m., 112°; in 20 m. more, 112½°; 1 hour after death, 113°. The body was now stripped, and laid out on a board, and covered with muslin; the axilla had been freely exposed, but gave in 20 m., 112°; at 7 h. and 40 m. P. M., it gave over 112°; at 7¾, 112°; at 7 h. 55 m., 111½°; at 8½, 111°; at 8¾, 110¼°; at 9, 109¾°; at 9¼, 109°, when the observations ended.

I have had much experience in this malady, but never happened to take my thermometer with me before; but, I am convinced that solar asphyxia, in the first degree, is the hottest of all maladies, and can hardly be less than 120° in many cases! The patient seldom has more than *one premonitory symptom; a sudden dryness and heat of the skin.* I suppose that this man's heat was below the average; certainly not so great as some others, according to my touch. Solar asphyxia, is, I believe, always fatal; its average duration is less than an hour, probably,—half that time will be nigher the truth. It is grievous to think how little discrimination is made in solar diseases;—what can be more different in symptoms and treatment than solar asphyxia? Solar exhaustion or syncope? Solar hyperæmia or excitement, etc. etc.? All forms but the first are readily cured, and even that might, by bloodletting, be cured or prevented, in all probability, in one or two minutes after the patient feels his skin becoming hot and dry, or after he falls, as happened in a case at my door; but, in a short time after the attack, the lancet is useless, or rather, hastens strangulation.

The temperature of the 23d of July, being the day previous to Jennings' death: At 6½ A. M., 84°; at 7, 85°; at 7½, 87°; at 8, 88°; at 9, 90°; at 10, (some haze) in the sun near a wooden wall, 115°; touching the same, 130°; at 11, sand in the street, 143°; at 2 P. M., near the sun, 150°; sand in the street, 152°; roof of a house, touching, 150°; at 8½, air 89°. River at 8 A. M., 83¾°; 3 P. M., 84½°; in the shade, near the sun, at 3, 102°; in the house, 97°; do. 4, 96°. Such is the history of this unexampled day, in which it is supposed 15 victims perished from insolation.

